# Commentary on: Noninvasive assessment of liver fibrosis using aspartate transaminase to platelet ratio index (APRI) in adult patients with chronic liver disease

**Published:** 2011-05-01

**Authors:** Mortada El-Shabrawi, Mona Isa

**Affiliations:** 1 Pediatric Hepatology Department, Cairo University, Cairo, Egypt

**Keywords:** Chronic hepatitis C, Fatty liver, Aspartate aminotransferases

Dear Editor,

We enjoyed reading the excellent article by Yilmaz and colleagues on noninvasive assessment of liver fibrosis using aspartate transaminase to platelet ratio index (APRI) in adult patients with chronic liver disease (CLD) [1]. They performed their tests on adults with chronic hepatitis C (CHC), B (CHB), and non-alcoholic fatty liver disease (NAFLD). We definitely need to develop serological markers that have satisfactory sensitivity, specificity, and high predictive values, which can be used either instead of liver biopsy or to reduce the frequency of needed biopsies for monitoring the evolution of CHC and defining the right moment for commencing treatment. Despite the study results showing that APRI has an acceptable accuracy for the assessment of liver fibrosis in adults with CHC and NAFLD, this was not the case in CHB patients. We believe that the study results would have been more valid if the researchers have used a combination of non-invasive tests to assess liver fibrosis. A similar study conducted in Hungary used APRI and liver stiffness (LS) measurements to assess fibrosis in CHC [2]. The combination of both fibrosis markers was useful for non-invasive assessment of fibrosis in CHC. Forestier and colleagues found that LS measurements, APRI score, (13)C-amniopyrine breath test and indocyanine green plasma clearance were reliable markers for assessing cirrhosis in patients with CLD [3]. APRI was also found to be a simple and readily available tool for assessing liver fibrosis in patients with biliary atresia during post-operative follow-up care [4].

A drawback for using APRI is that its performance is not reliable in women, younger patients and in those with non-vertically-transmitted hepatitis C virus infection [5]. Alternatively, our group recommends the use of Fibrotest (FT) as a non-invasive serum biomarker for the assessment of the degree of hepatic fibrosis in pediatric patients with CHC. FT at a cutoff value of 0.25 was able to discriminate patients with mild stage of fibrosis from those with no or minimal fibrosis (sensitivity of 92.3%, specificity of 95.8% and accuracy of 94%). A higher cutoff point (FT = 0.54) can be used to diagnose significant fibrosis (ie., moderate or severe stages) with a sensitivity of 71.4%, specificity of 90.7%, and accuracy of 88.0% [6].
